# Adding flexibility to clinical trial designs: an example-based guide to the practical use of adaptive designs

**DOI:** 10.1186/s12916-020-01808-2

**Published:** 2020-11-19

**Authors:** Thomas Burnett, Pavel Mozgunov, Philip Pallmann, Sofia S. Villar, Graham M. Wheeler, Thomas Jaki

**Affiliations:** 1 Department of Mathematics and Statistics, Lancaster University, Fylde College, Lancaster, LA1 4YF UK; 2 Centre for Trials Research, College of Biomedical & Life Sciences, Cardiff University, Cardiff, UK; 3 MRC Biostatistics Unit, University of Cambridge School of Clinical Medicine, Cambridge Institute of Public Health, Forvie Site, Robinson Way, Cambridge Biomedical Campus, Cambridge, CB2 0SR UK; 4 Cancer Research UK & UCL Cancer Trials Centre, University College London, 90 Tottenham Court Road, London, W1T 4TJ UK

**Keywords:** Novel designs, Innovative trials, Efficient methods, Enrichment designs, Multi-arm multi-stage platform trials

## Abstract

Adaptive designs for clinical trials permit alterations to a study in response to accumulating data in order to make trials more flexible, ethical, and efficient. These benefits are achieved while preserving the integrity and validity of the trial, through the pre-specification and proper adjustment for the possible alterations during the course of the trial. Despite much research in the statistical literature highlighting the potential advantages of adaptive designs over traditional fixed designs, the uptake of such methods in clinical research has been slow. One major reason for this is that different adaptations to trial designs, as well as their advantages and limitations, remain unfamiliar to large parts of the clinical community. The aim of this paper is to clarify where adaptive designs can be used to address specific questions of scientific interest; we introduce the main features of adaptive designs and commonly used terminology, highlighting their utility and pitfalls, and illustrate their use through case studies of adaptive trials ranging from early-phase dose escalation to confirmatory phase III studies.

## What are adaptive designs?

In a traditional clinical trial, the design is fixed in advance, the study is carried out, and the data analysed after completion [1]. In contrast, adaptive designs pre-plan possible modifications on the basis of the data accumulating over the course of the trial as part of the trial protocol [2]. We consider designs that allow for modifications of the trial such as the sample size, the number of treatments, or the allocation ratio to different arms. We do not consider options such as stopping early due to failure to meet operational criteria or excessive safety events, although adaptive designs for some of these do also exist [3]. Adaptive design methodology has been around for more than 25 years [4], with some methods such as group sequential designs being even older [5].

It is crucial that the adaptive nature of a design does not undermine the trial’s integrity and validity [6]. By integrity of a trial, we mean that the data have not been used in such a way as to substantially alter the result, while the validity of the results requires that the study answers the original research questions appropriately. Adaptive designs require procedures to ensure that data is collected, analysed, and stored in an appropriate manner at every stage of the trial, with specialised statistical methodology for inference. The involved logistical and statistical nature of adaptive designs should also be reflected in their reporting [7].

Flexibility of a design is not a virtue in itself but rather a gateway to more efficient and ethical trials where futile treatments may be dropped sooner, more patients may receive a superior treatment, fewer patients may be required overall, treatment effects may be estimated with greater precision, a definitive conclusion may be reached earlier, etc. Adaptive designs can aid in these aspects across all phases of clinical development [2].

Despite the many clear benefits, many modern adaptive designs are still far from established as typical practice [8–10]. Many reasons for this have been identified [9–12], the main of which include the following: lack of expertise and experience (in the application of adaptive designs among clinicians, trialists, and statisticians), lack of design and analysis software, time required for planning and analysis, inadequate funding structure to account for design uncertainty, and the fact that chief investigators may prefer more familiar methods. We believe that the main reason investigators are not inclined to adopt adaptive designs is a lack of clarity about what these are and what they can (and cannot) accomplish and how they may be implemented. Ambiguous terminology and vague definitions add to this confusion [13], and hence, we provide a glossary of common types of adaptive design in Table 1. Other work providing reviews of recent uses of adaptive designs may provide insight into designs not covered in detail here [4, 14].

**Table 1:** Glossary of adaptive designs and descriptions of their typical applications

Method	a.k.a	Phase of development	Definition	Targeted benefits
**What is a safe dose?**				
Continual re-assessment method	CRM	I	Dose-escalation design for finding the maximum tolerated dose (MTD)	More accurate and precise estimation of the MTD than with 3+3 designs, more patients treated at or close to the MTD
Escalation with overdose control	EWOC	I	Dose-escalation design to find MTD using an allocation criterion to avoid overdosing	More accurate and precise estimation of the MTD than with 3+3 designs, avoiding undesirable overdosing of patients
**Which is the best treatment of multiple options?**				
Adaptive treatment switching		II/III	Allow trial participants to switch from allocated treatment to an alternative	More trial participants receive preferred treatment
Drop the loser	DTL	II/III	Drop inferior treatment arms (control group typically retained)	Fewer trial participants assigned to less effective treatments
Multi-arm multi-stage trial	MAMS	II/III	Compare multiple treatments to a common control, allow for early stopping for efficacy or futility	Common control requires fewer patients than conducting separate trials, early stopping for efficacy or futility
MCP-Mod		II	Combination of multiple comparisons and modelling approaches to establish dose-response model	Efficient use of available data vs pairwise comparisons
Response-adaptive randomisation	Adaptive allocation, RAR	II/III	Shift allocation ratio towards more promising treatment(s)	More trial participants receive effective treatment
**Which patients will benefit?**				
Basket trials			Examine a single experimental treatment in multiple sub-types of a biomarker	Identify and target biomarker sub-types that benefit from the treatment
Biomarker adaptive	Adaptive signature	II/III	Identify and utilise biomarker information to modify trial in progress to target population	Target the correct patient population
Covariate-adjusted response adaptive	CARA	II/III	Shift allocation ratio towards promising treatment(s) using covariate information	More trial participants receive effective treatment
Population enrichment	Adaptive enrichment	II/III	Allow for selection of target population during the trial based on pre-defined patient populations	Target the correct patient population
Umbrella trials			Multiple biomarkers each paired with specific treatments	Target the appropriate to treatment within each patient group
**Does the treatment work?**				
Group sequential design		II/III	Early stopping for futility or efficacy	Reduction in the expected sample size, typically allowing for faster trials requiring fewer patients (for a small increase in the possible maximum sample size)
Sample size re-assessment	Sample size re-estimation/re-calculation	II/III	Mid-course adjustment of the sample size, in either a blinded or unblinded fashion	Raise the probability of a successful trial
**Broader topics in adaptive designs**				
Bayesian adaptive		I/II/III	Bayesian methodology may be incorporated into many other designs in the analysis and/or the interim decision-making	Lower sample size due to utilisation of prior information
Seamless design	Portfolio decision-making	I/II/III	Merge trials from different phases of development, e.g. phase I/II or phase II/III, can be inferentially and/or operationally seamless	(Inferential) More efficient use of data from each phase of clinical development/(operational) faster clinical development process and moving between stages

To demonstrate how and when adaptive designs can be useful, we focus on four key questions of scientific interest when developing and testing novel treatments: ‘What is a safe dose?’ ‘Which is the best treatment among multiple options?’ ‘Which patients will benefit?’ ‘Does the treatment work?’ For each of these questions, we briefly review several important adaptive designs, outlining their advantages and disadvantages. We illustrate their application through real-world examples.

## When to use an adaptive design

### What is a safe dose?

Phase I trials of new drugs are conducted to assess the safety of a treatment, the aim being to establish the safety profile across a range of available doses, in order to select a dose for further testing. In many therapeutic areas, the goal is to identify the maximum tolerated dose (MTD), that is the highest dose that controls the risk of unacceptable side effects [15] and hence is deemed safe. In practice, one seeks to identify the dose at which the probability of a dose-limiting toxicity (DLT) is equal to some pre-specified target level, usually around 20–33%. This is done by treating consenting patients sequentially at increasing doses until too high a proportion of unacceptable side effects are observed.

#### 3+3 design

The most commonly used method for conducting dose-escalation studies in oncology is the 3+3 design [16, 17]. It is a simple, rule-based approach under which patients are dosed in cohorts of three. Based on the number of DLTs observed in the current cohort of patients, recommendations are made to dose the next three patients at either the next escalating dose or the current dose. Upon observing a pre-specified number of toxic outcomes at a dose level (say DLTs in more than 2 in 6 patients), the trial is terminated and the dose level below is considered to be the MTD. The 3+3 design is a special case of the more general A+B design [18]; when a new dose is introduced, a cohort of A patients are dosed, and if further observations are required on the same dose, a cohort of B further patients are then dosed.

**Example** Park et al. [19] performed a phase I dose-escalating study of docetaxel in combination with 5-day continuous infusion of 5-fluorouracil (5-FU) in patients with advanced gastric cancer. The study used a 3+3 design to find the MTD from four dose levels of 5-FU. The treatment consisted of docetaxel 75 mg/m^2^ on day 1 in a 1-hour infusion followed by 5-FU in continuous infusion from day 1 to day 5, according to the escalating dose levels. The starting dose of 5-FU was 250 mg/m^2^/day for 5 days. In the absence of any DLTs (defined as febrile neutropenia and/or grade 3/4 toxicity of any other kind apart from alopecia), dose escalation in additional cohorts continued, increasing the dose by 250 mg/m^2^/day for each increment.

The first DLT was observed at dose level 2 (5-FU 500mg/m^2^/day for 5 days). Three additional patients were enrolled at this dose level, none of whom experienced any DLT. Thus, dose escalation proceeded to dose level 3 (5-FU 750 mg/m^2^/day for 5 days) where a further 2 patients experienced DLTs and so dose escalation was stopped. Dose level 2 was therefore the recommended regimen with docetaxel 75 mg/m^2^ on day 1 and 5-FU 500 mg/m^2^/day in a 5-day continuous infusion.

**Advantages** The key advantage of the 3+3 design is that it does not require any time to design. In addition, this method is well-known to clinicians, often leading to its use being well motivated within the trial team. Web applications [20] are available to understand the performance of such designs.

**Disadvantages** The major disadvantages of the 3+3 design will become clear as we draw comparisons to the methods that follow. In particular, we note that the MTD is not explicitly defined; this means that the most likely dose to be chosen as the MTD can have a probability of DLT far from the assumed target and can be highly variable [21–23].

Rule-based dose-escalation methods such as 3+3 designs are seriously flawed, which runs afoul of the part of our definition of an adaptive design that demands integrity and validity. Thus, this method being well-known to clinicians, possibly allowing them to avoid collaboration with a statistician, can also present a serious problem.

#### Continual re-assessment method

The continual re-assessment method (CRM) [24, 25] models the relationship between dose and the risk of a patient experiencing a DLT, using an iterative process to make use of all available trial data when choosing the dose for the next patient cohort. Based on all available data from the trial, the relationship between dose and toxicity is modelled to inform the choice of dose for the next cohort. The dose for the next patient or cohort is chosen as either that with an estimated probability of DLT closest to the target toxicity level, or the highest available dose below the target level. This process is iterated for each new cohort of patients, ensuring that at all times all available data are used. The application of the CRM process is highly flexible, allowing the investigators to adjust the design to suit the particular trial and trialist (making use of all trial data wherever it is introduced, as is seen in the example to follow). Both the cohort size and the sample size of a CRM trial are determined before the trial begins; sample sizes are often planned with practical constraints in mind rather than statistical properties while simulation may be used to understand statistical operating characteristics [26].

**Example** Paoletti et al. [27] provide a tutorial of the practical considerations for designing CRM trials; they describe the design, conduct, and analysis of a multicentre phase I trial to find the MTD (defined as the dose with probability of a DLT closest to 20%) of rViscumin in patients with solid tumours. A DLT was defined as any haematological grade 4 or non-haematological grade 3 or grade 4 adverse event as defined by the National Cancer Institutes Common Terminology Criteria for Adverse Events (NCI CTCAE) Version 2, with the exclusion of nausea, vomiting, or rapidly controllable fever. The starting dose of the trial was 10 ng/kg, with fixed dose levels for further exploration of 20, 40, 100, 200, 400, and 800 ng/kg; if no adverse events of grade 2 or higher were observed after escalation to 800 ng/kg, additional doses would be added in increments of 800 ng/kg (i.e. 1600, 2400 ng/kg).

The trial used a two-stage CRM design [24] allowing the low doses to be rapidly moved through while utilising the model-based approach in the selection of the MTD. During the first stage, one patient was assigned to the starting dose of 10 ng/kg, and if adverse events were absent or grade 1, a new patient was given the next highest dose; if a non-DLT adverse event of grade 2 or higher was observed, a further two patients would be given the same dose. Escalation continued in this manner until the first DLT was observed, at which point the model-based design took over. A one-parameter model [25] was fitted to the data, and the dose with an estimated probability of DLT closest to 20% was recommended for the next patient, subject to the constraint that no untested dose level is skipped. The trial was stopped when the probability of the next five patients being given the same dose was at least 90% (i.e. the trial would be unlikely to gain further information that would affect dose allocation).

The first DLT was observed in the 11th patient who was given 4000 ng/kg, at which point the CRM part of the design took over. In total, 37 patients were recruited to the trial before it was terminated under the aforementioned rule, and the MTD declared as 5600 ng/kg, with an estimated DLT probability of 16%; the estimated probability of a DLT at the next highest dose (6400 ng/kg) was 31%. It is worth noting that during the ongoing trial, the first DLT was recoded to a non-DLT; this change is easily incorporated in a CRM design by simply re-estimating the DLT risks at each dose using the updated data [26]. This recoding of the first DLT had an impact on the overall trial outcome as without this a lower MTD would have been selected [27]. This illustrates one of the benefits of a model-based approach; deviations from the planned course of the trial are handled without compromising the validity of the design.

The authors describe how the statistical work of the trial helped to inform study clinicians and the Trial Steering Committee, with whom any final decisions rest. For example, a decision was made to dose another patient at 3200 ng/kg rather than escalate to 4000 ng/kg as per the design in order to gather more PK data at this level. Furthermore, 10 extremely tolerable (but presumably inefficacious) dose levels were cleared quickly and with far fewer patients than the 3+3 design would require.

**Advantages** Conceptually, the CRM is a far wiser approach (and more efficient/economic) than the 3+3 design because it uses all available trial data to make decisions, rather than solely the data from the last cohort [28, 29]; the CRM also targets a pre-specified toxicity level. Numerous comparative simulation studies have shown the CRM to supersede the 3+3 design by dosing more patients in trial at or near the correct MTD and also by selecting the correct dose as the MTD more often [30–34], which in turn can result in higher probability of success in subsequent phase II and phase III clinical trials [35].

Furthermore, the CRM can easily be adapted to include more informative endpoints as follows: multiple graded toxicities to incorporate severity of side effects [36–38], combinations of safety and efficacy [39–44], time-to-event outcomes to distinguish between toxicity events occurring sooner or later [45, 46], or even developed to escalate multiple treatments at once [47–54]. Regulatory authorities are also recognising that novel adaptive designs using statistical models are of great importance, and actively encourage sensible usage of them in phase I trials [55, 56].

**Disadvantages** The main disadvantage of the CRM design is the time and effort required at the design stage to assess how the trial is expected to perform. This requires close collaboration between the clinical team and a suitably trained statistician who is able to guide this optimisation process, although this opportunity to consider the design more carefully can only be a good thing. The clinical team may still see the CRM as a ‘black box’; to resolve this concern, Dose Transition Pathways provide a tool for visualisation of the CRM escalation/de-escalation decisions [57]. Several computer programs are available (see MD Anderson Cancer Center software library [58], Vanderbilt University, and packages within R [59, 60]) for conducting simulation studies, some of which can offer comparisons to other popular dose-escalation designs [60] and tutorial papers are available offering further guidance [26]. Web-based solutions are on offer for both the CRM and conceptual equivalents [61–63].

#### Escalation with overdose control

The escalation with overdose control (EWOC) approach [64] is similar to the CRM in that all available data are used to make dose-escalation decisions with a target toxicity level used to choose which dose level the next patient or cohort should receive. However, the EWOC approach assigns the next patient using a skewed allocation criterion to account for the fact that the overdosing of patients is much more undesirable compared to the underdosing. This results in a more conservative patient allocation approach, with fewer patients being exposed to possible overdosing compared to the CRM, while still benefiting from the model attempting to allocate the patients near the MTD [65, 66]. Additionally, the same statistical model is re-expressed in a way that allows focus on the clinically relevant parameters, the MTD, and the probability of a DLT at the lowest dose. This means that prior information about the treatment being investigated can easily be incorporated and one can visualise how the distribution of the MTD changes over the course of the trial.

**Example** Nishio et al. [67] conducted a dose-escalation study of ceritinib in patients with advanced anaplastic lymphoma kinase-rearranged, non-small-cell lung cancer, or other tumours. A Bayesian EWOC approach was used to allocate the dose for the next patient. This allocates the next patient to the largest dose with an estimated probability of less than 25% that the risk of a DLT exceeds 33%. In total, 19 patients were recruited to the trial: three patients received doses of 300 mg, six patients received doses of 450 mg, four patients received doses of 600 mg, and six patients received doses of 750 mg. Two patients experienced DLTs, one at 600 mg and the other at 750 mg. The MTD was chosen as 750 mg, the largest dose at which the estimated probability of the risk of a DLT exceeding 33% was less than the target 25% (the probability was 7.3*%* for the chosen dose). Although the aim of the trial was not to evaluate efficacy of ceritinib in this population, 10 patients achieved partial responses to their cancers.

**Advantages** The EWOC approach offers a more cautious dose-escalation design that reduces the chance of patients being treated at excessively toxic doses [64]. Similar to the CRM, the EWOC approach has also been adapted to be used in trials with more complex outcomes, such as time-to-event data [68], and for combinations of treatments [69, 70]. Furthermore, the escalation control threshold can be altered depending on the trial context and may change during the conduct of the trial [65, 66]; this offers a conservative dose-escalation schema at the start of the trial when there is little data available, but as more data are accrued, dose-escalation gradually becomes less conservative, and the MTD can be targeted more quickly than with the standard EWOC approach [65, 66, 71].

**Disadvantages** A slower dose-escalation approach may increase the number of patients treated at sub-therapeutic doses. Similar to implementation of the CRM, care is required when designing trials using the EWOC approach. For example, choice of the MTD estimator needs to be considered; several trials use the same criterion as that by Nishio et al. [67], i.e. the MTD is the dose that would be given to a new patient had they entered a trial. The implications of each choice need to be considered well in advance [72]. Furthermore, if the investigators plan to relax the escalation control mechanism, as has been done in practice before [66, 73, 74], the implications of this decision need to be considered. The EWOC approach may recommend to escalate the dose even when the most recently evaluated patient experienced a DLT [75].

#### Summary

Despite the 3+3 designs frequent use in phase I clinical trials over the last 30 years [8, 76–78], there is overwhelming consensus among statisticians and methodologists that it is sub-optimal, and more efficient designs for identifying the MTD should be used [35, 79, 80]. Many alternative designs propose the use of statistical models, such as the two alternatives we have presented here; both of which have superior operating characteristics over the 3+3 design.

The model-based approaches above serve as the main framework for other proposed approaches designed for trials with novel drug combinations, endpoints that use time-to-event data and/or efficacy outcomes, or information about the severity of observed toxicities. These designs have found their way into clinical practice in recent years, primarily in oncology for cytotoxic treatments. However, they can be used for novel molecularly targeted anti-cancer therapies [81], and in other disease areas altogether: O’Quigley et al. [82] proposed CRM-type designs for anti-retroviral drugs to treat human immunodeficiency virus (HIV), Lu et al. [83] conducted a dose-escalation study of quercetin in patients with hepatitis C; Whitehead et al. [84] proposed a model-based design for trials in healthy volunteers, and Lyden et al. [85] used a CRM design in the RHAPSODY trial in stroke patients. Combining the advantages of both model-based and rule-based designs can also be desirable, with proposals such as the Bayesian optimal interval (BOIN) design [86, 87].

There is no ‘one size fits all’ approach for conducting adaptive dose-escalation studies, but there is overwhelming evidence that model-based designs are far better than rule-based designs, such as the 3+3. Model-based designs are on the whole more efficient in their use of data, less likely to dose patients at sub-therapeutic doses, more likely to recommend the correct MTD at the end of the trial, and provide an MTD estimate that directly relates to a specified target level of toxicity. We have discussed two approaches here for brevity, though many other alternatives have also been proposed, including designs based on optimal design theory [88–91] and model-free designs [86, 92–94], without the shortcomings of common rule-based designs like 3+3. The increasing usage of model-based designs in practice, as well as their acknowledgement in regulatory guidance and the provision of guideline documents [95], formal courses and computer software is indicative of the changing tide of clinical practice for phase I trials. Although the fact remains that such designs are more complex making implementation more challenging, for a trained statistician unfamiliar with the design planning, such a trial would require a significant investment of time. Such issues in implementing novel statistical methods are well recognised [96].

### Which is the best treatment among multiple options?

After establishing the safety of a treatment, we next examine its efficacy. In this section, we consider randomised clinical trials that aim to select the best treatment among multiple experimental treatment arms (these can be different treatments, doses of the same treatment, or combinations of the two). The methods we explore are typically considered for use in phase II of the development process, where we wish to select a treatment or dose for further study. We explore methods that seek to remove less beneficial treatments from the trial quickly, giving patients a better chance of receiving an efficacious treatment. In trials where the different arms correspond to a series of exposure levels (such as doses of a drug, duration or intensity of radiotherapy, or number of therapy sessions), model-based approaches examine the dose-response relationship to provide a deeper understanding of this in an efficient way.

#### Multi-arm multi-stage

**Fig. 1:**
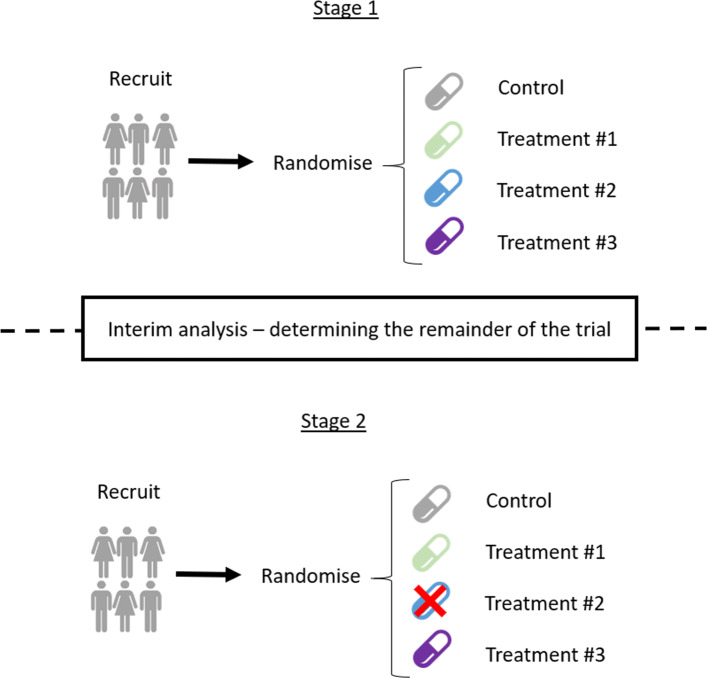
A two-stage four-arm MAMS design

Multi-arm multi-stage (MAMS) [97] trials allow the simultaneous comparison of multiple experimental treatment arms with a single common control. They are conducted over multiple stages: allowing for the early stopping of recruitment for either efficacy or futility. For example, if an experimental treatment is found to be performing poorly, it may be dropped for futility at a pre-planned interim analysis (if all experimental arms are dropped, the trial is stopped for futility); alternatively, the trial may end early when a treatment is shown to be sufficiently efficacious. We cover group sequential designs in more detail in the ‘Group sequential designs’ section, but MAMS designs apply similar methodology while testing multiple experimental treatments simultaneously. A simplex schematic of how a two-stage four-arm trial using MAMS design can progress is given in Fig. 1.

MAMS trials are designed using a pre-planned set of adaptation rules to find the best treatment to carry forward for further study [98] or carry forward all promising treatments [99]. Alternatively, more flexible testing methods [100–102] allow methodological freedom as to how adaptation decisions are made. Open source software is available to assist in the design and analysis of MAMS trials in the form of the ‘MAMS’ package for R [103]. Alternatively, in STATA, there are several modules available such as ‘nStage’ [104], ‘nStagebin’ [105], and ‘DESMA’ [106].

**Example** The TAILoR trial [107] was a phase II, multicentre, randomised, open-labelled, dose-ranging trial of telmisartan using a two-stage MAMS design. The trial planned recruitment of up to 336 HIV-positive individuals over a 48-week period, with a single interim analysis planned after 168 patients had completed 24 weeks on either an intervention or a control treatment. Patients were randomised with equal probability to one of four groups: no treatment (control), 20 mg telmisartan daily, 40 mg telmisartan daily, or 80 mg telmisartan daily.

At the interim analysis, there were three possible outcomes based on assessment of change in HOMA-IR index from baseline to 24 weeks: if one telmisartan dose was substantially more effective than control, the study would stop and that dose would be recommended for further study; if all telmisartan doses were less effective than control, the study would stop with no dose recommended for further study; if one or more doses were better than control but none met the first criterion, the study would continue and patients would have been randomised between these remaining dose(s) and control. If a second stage was conducted, then a final analysis would be conducted with two possible outcomes: either the best dose is significantly more effective than the control in which case it is recommended for further study, or no dose is significantly better than control in which case no dose is recommended.

A total of 377 patients were recruited [108] (note this difference in sample size was due to higher than expected dropout). In stage 1, 48, 49, 47, and 45 patients were randomised to control and 20, 40, and 80 mg telmisartan, respectively. At the interim analysis, the 20- and 40-mg telmisartan groups performed worse than control on average and so only 80 mg telmisartan was taken forward into stage 2. At the end of stage 2, 105 patients had been recruited to control and 106 to the 80-mg arm (in total); there was no difference in HOMA-IR (estimated effect, 0.007; SE, 0.106) at 24 weeks between the telmisartan (80 mg) and control arm. If a traditional fixed sample design had been used in place of a MAMS design, all experimental arms would have been studied throughout the trial, requiring a further 100 or so patients for arms that ultimately did not demonstrate an effect of the experimental treatment.

**Advantages** MAMS designs are useful when there are multiple promising treatments with no strong belief that one treatment will be more beneficial. The use of a shared control group considerably reduces the number of patients that need to be recruited compared to separate RCTs testing each treatment. Other advantages are as follows: treatments that provide no benefit to patients are dropped from the trial; patients have a higher chance of receiving an experimental treatment compared to a 2-arm trial, which may improve recruitment to the study [109, 110]; administratively and logistically, effort is only required for one trial and thus can substantially speed up the development process [111].

**Disadvantages** MAMS trials require an outcome measure that allows a timely decision about the worth of each treatment. Consequently, the primary endpoint needs to be relatively quickly observed (in comparison to patient accrual) or an intermediate measure that is strongly associated with the primary endpoint is required for interim decision-making. MAMS designs require a larger potential maximum sample size than a corresponding multi-arm fixed design (although smaller than several separate trials). The MAMS approach has a variable sample size depending on which decisions are made during the trial, making planning more cumbersome, although the possible pathways are pre-defined; this is more variable than is typical of even other adaptive designs because decisions relate to each treatment individually.

#### Drop the loser

Drop the loser (DTL) designs [112, 113] are closely related to MAMS designs [114] in that they compare several experimental treatments to a common control over multiple stages. The key difference is that in a DTL design, it is pre-determined how many arms will be dropped after each stage of the trial. As the name suggests, the worst performing experimental treatments are dropped at interim analysis, leaving only one treatment to compare to control at the final analysis.

**Example** The ELEFANT trial [115] is a randomised controlled, multicentre, three-armed trial testing whether early elimination of triglycerides and toxic free fatty acids from the blood is beneficial in HyperTriGlyceridemia-induced Acute Pancreatitis (HTG-AP). The study uses a two-stage DTL design; in the first stage, patients with HTG-AP are randomised with equal probability into three groups: patients who undergo plasmapheresis and receive aggressive fluid resuscitation, patients who receive insulin and heparin treatment with aggressive fluid resuscitation, and patients with aggressive fluid resuscitation only (the control). At the interim analysis, the two experimental treatments will be compared and the one demonstrated to be the best will be retained for the remainder of the trial. Thus, in the second stage, patients are randomised into two groups, the control and the selected experimental treatment. At the conclusion of the trial, formal statistical comparisons may be drawn between the control and the experimental treatment selected at the interim analysis. The target sample size is 495 in order to detect a 66% relative risk reduction, using a 10% dropout rate with 80% power at 5% significance level. The study began in February 2019 and is expected to finish December 2024.

**Advantages** As with MAMS designs, the key advantage is the use of the shared control group which reduces the number of patients required. Of further practical benefit is the guarantee that a pre-specified number of arms will be dropped during the course of the trial, meaning that the required sample size is known before recruitment begins [114]. At the conclusion of the trial, only one experimental treatment remains to be compared against the control making for a clear interpretation of results.

**Disadvantages** The choice to only continue the most efficacious treatments may cause concern; consider for example an interim analysis where a dropped treatment has demonstrated almost equivalent performance to a treatment that continues in the trial, it is possible that a suitable treatment has been dropped by chance. In addition, there is a similar operational complexity to MAMS designs as it is unknown which treatments will be carried through the trial.

#### Response-adaptive randomisation

**Fig. 2:**
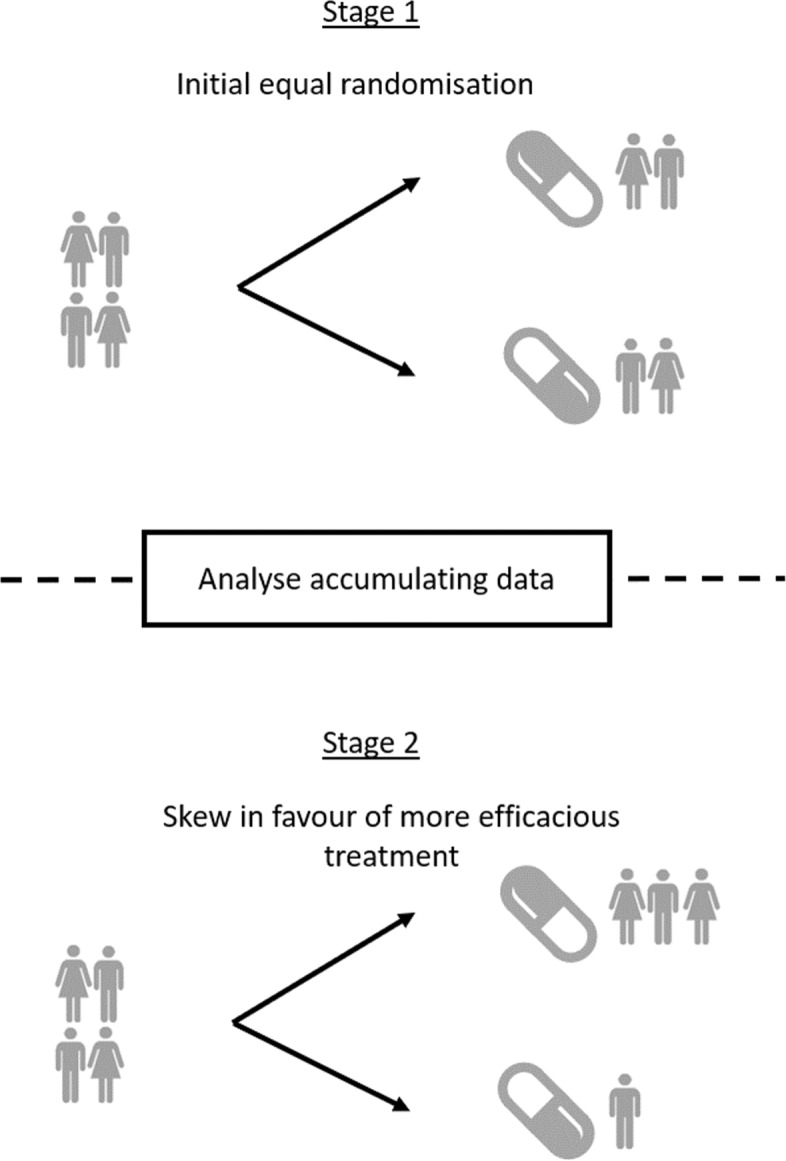
A description of a RAR procedure

At the beginning of the trial, the comparable performance of the experimental treatment arms may be unknown; hence, equal randomisation is sensible under a clinical equipoise principle. However, as data accumulates, it becomes challenging from an ethical standpoint to randomise patients to a treatment arm that data suggest may be inferior. To resolve this, response-adaptive randomisation (RAR) aligns the randomisation probabilities with the observed efficacy of the different arms (Fig. 2).

RAR dates back to 1933 [116], and since then, several methods to align randomisation probabilities and observed evidence of efficacy have been proposed [117, 118]. Thompson [116] proposed to randomise patients to arms with a probability that is proportional to the probability of an arm being the best arm. Regardless of how these probabilities are defined and applied, they can be also used to define further adaptations to the trial depending on the values they assume. For example, if the allocation probability goes below or rises above a certain value, arms can be dropped for futility or selected similar to a MAMS study [119, 120]. Free software is available from the MD Anderson website [58]. The R package ‘bandit’ offers an alternative to the implementation of such designs [121].

**Example** A prospective, randomised study reported by Giles et al. [122] was conducted in patients aged 50 years or older with untreated, adverse karyotype, acute myeloid leukaemia to assess three troxacitabine-based regimens: idarubicin and cytarabine (the control arm), troxacitabine and cytarabine, and troxacitabine and idarubicin. The trial used a Bayesian RAR design along the lines proposed by Thompson [116]. Thirty-four patients were recruited and randomised to one of the three arms. Initially, there was an equal chance of randomisation to each arm. The randomisation probabilities were updated after every patient such that treatment arms with a higher success rate, defined as the proportion of patients having complete remission within 49 days of starting treatment, would receive a greater proportion of patients. The design would drop arms if their assignment probabilities became too low or promote them to phase III if their assignment probability was high enough. The probability of a patient being randomised to the control arm was fixed until the first experimental arm was dropped. This occurred when the randomisation probability for the dropped experimental arm was 0.072 [123].

Of the thirty-four patients recruited, 18 were randomised to idarubicin and cytarabine, randomisation to troxacitabine and idarubicin stopped after five patients, and randomisation to troxacitabine and cytarabine stopped after 11 patients. Success rates were 55% (10 of 18 patients) with idarubicin and cytarabine, 27% (three of 11 patients) with troxacitabine and cytarabine, and 0% (zero of five patients) with troxacitabine and idarubicin.

**Advantages** RAR can increase the overall proportion of patients enrolled in the trial who benefit from the treatment they receive while controlling the statistical operating characteristics [124–126]. This mitigates potential ethical conflicts [127] that can arise during a trial when equipoise is broken by accumulating evidence and makes the trial more appealing to patients [110] possibly improving trial recruitment [128]. The main motivation for RAR designs is to ensure that more trial participants receive the best treatments; it is possible to use such methods to optimise other characteristics of the trial [129]. In a multi-armed context, RAR can shorten the development time and more efficiently identify responding patient populations [130].

**Disadvantages** RAR designs have been criticised for a number of reasons [131] although many of the raised concerns can be addressed. Logistics of trial conduct is a noticeable obstacle in RAR due to the constantly changing randomisation [132]; requiring more complex randomisation systems may in turn impact things such as drug supply and manufacture. When the main advantage pursued is patient benefit, this may compromise other characteristics; for example, a two-arm RAR trial will require larger sample sizes than a traditional fixed design with equal sample sizes in both arms; methods to account for such compromise have been proposed [124, 133].

Choosing an approach from the variants of RAR can be challenging; in most cases, balancing the statistical operating characteristics and randomly assigning patients in an ethical manner is required. Most RAR methods require the availability of a reliable short-term outcome (although the exact form of the data may vary [124, 134]); however, this can result in bias, requiring the use of extra correction methods for estimation purposes [135]. Another statistical concern is control of the type I and type II error rates. As discussed above, this is possible but requires intensive simulations or the use of specific theoretical results [118, 129, 130, 136]; this creates an additional burden at the design stage, requiring additional time and support.

#### Multiple comparison procedures and modeling approaches (MCP-Mod)

In phase II dose-ranging studies, patients are typically randomised to either one of a number of doses and possibly a placebo. The target dose is often the minimum effective dose, the smallest dose giving a particular clinically relevant effect. A traditional approach to find the target dose is based on pairwise comparisons. However, this only uses the information from the corresponding doses and typically results in larger sample sizes required in the trial [137]. As an alternative, MCP-Mod [138, 139] employs a dose-response model allowing for interpolation between the doses.

**Fig. 3:**
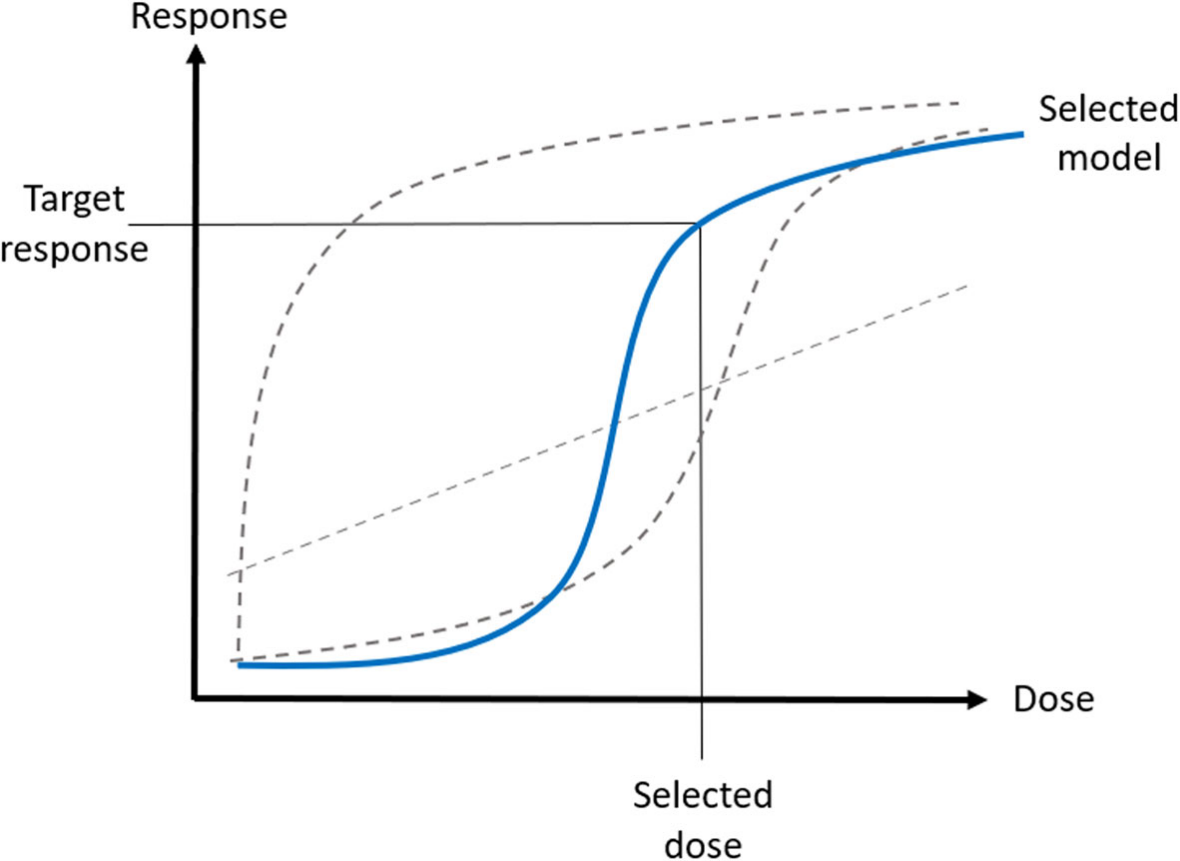
Model fitting in MCP-Mod

MCP-Mod is a two-stage method that combines Multiple Comparison Procedures (of dose levels) and MODeling approaches. At the planning stage, the set of possible models for the relationship between dose and response are defined, such as those shown in Fig. 3. The inclusion of several models addresses the issue of some of the models being mis-specified. At the trial stage, the MCP step checks whether there is any dose-response signal. This is done through hypothesis tests for each model, adjusting for the fact that there are multiple candidate models. This controls the probability of making an incorrect claim of a dose-response signal (a type I error). If no models are found to be significant, it is concluded that the dose-response signal cannot be detected given the observed data.

With a dose-response signal established, a single model is selected, or if multiple models are selected, an average is made. The selection of models can be based either on tests performed at the MCP step or on some other measures such as information criterion [140]. The chosen model is used to select the best dose. We refer the reader to the works focusing on the step-by-step application of MCP-Mod in practice [137, 141].

**Example** Verrier et al. [142] described the application of MCP-Mod in a placebo-controlled parallel group study undertaken in hypercholesterolaemic patients, which evaluated the change in low-density lipoprotein cholesterol (LDLC, mg/dL) following 12 weeks treatment as the primary endpoint. Three active doses were studied: 50, 100, and 150 mg, and nearly 30 patients per treatment group were recruited. The objective of the trial was twofold: (i) to demonstrate the dose effect of the compound and (ii) to select the dose providing at least a 50% decrease in LDLC.

The set of candidates was composed of linear, logistic (four possible pairs of parameters obtained from the guess that 50% of the maximum effect occurred at 50, 75, 100, or 125 mg, respectively, defined four models), and quadratic (corresponding to the maximum effect at 125 mg) models. The hypothesis tests selected a logistic model, and the estimated target dose was 76.7 mg. Alternatively, an information criterion approach selected the quadratic model and the estimated target dose was 78.2 mg. To check the robustness of the results, model averaging was used and resulted in nearly the same estimated target dose. This analysis informed the selection of the dose for phase III trials for which the dose of 75 mg was chosen. This choice of dose was not one of those three active doses directly studied but could be selected due to using a model-based approach.

**Advantages** MCP-Mod allows for a more efficient use of data. There are many practical recommendations available [137], and it has been successfully applied in a number of trials [143]. The European Medicines Agency issued a qualification opinion of MCP-Mod [144] concluding that MCP-Mod uses available data better than the traditional pairwise comparisons, and the FDA also designated the method as fit for purpose [145]. There is software that implements the methodology, e.g. an R-package, DoseFinding [146] and PROC MCPMOD in SAS.

**Disadvantages** The method can be sensitive to the model assumptions [147], which can result in significantly lower power if the dose-response relationship is not well approximated by one of the pre-specified candidate models. The number of doses to be included should inform the choice of the candidate models. When the treatment regimens consist of various drugs and schedules (and doses within each), such disadvantages are amplified and MCP-Mod should be approached with much care.

#### Summary

The methods presented in this section are suitable for selecting a treatment or dose for further study and can allow for formal testing in a confirmatory setting. With RAR, we see the goal of focusing on the more effective treatments was thought of as an important topic almost 90 years ago; however, it is only in the last 30 years or so that this topic has gained traction as a more active area of methodological research. The knowledge on these more modern methods will need to be broadly shared before we start to see their use more widely in clinical practice [148].

Each of the methods discussed uses adaptation to make efficient comparisons of several treatments or doses. There is a common advantage of reducing the (expected) number of patients required to achieve the same strength of evidence when compared with fixed sampling alternatives. The key challenge is making design decisions given the uncertainty about how the trial will develop.

For RAR, MAMS, and DTL designs, the trial focuses on those treatments demonstrating effectiveness. These methods are appealing as they increase the chance of receiving a treatment that is more likely to be effective. Further to this, the model-based approach of MCP-Mod increases understanding of the relationship between dose and response in order to better allocate patients based on current trial data, in turn allowing greater confidence about the choice of dose.

### Which patients will benefit?

Late in the development cycle, such as phase III of drug development, we wish to confirm the treatment is effective. An important aspect of this is to ensure the right patients receive the treatment (i.e. those who will gain a meaningful benefit). Here, we focus on trials that use clinically relevant biomarkers to identify patients who may be sensitive to a treatment and therefore likely to respond.

#### Covariate-adjusted response adaptive

A form of RAR (see the “Response-adaptive randomisation” section) that accounts for patient differences is covariate-adjusted response adaptive (CARA). Randomisation probabilities are aligned to the patient’s observed biomarker information skewing allocation probabilities towards the best performing arms according to a patient’s set of characteristics. Such changes based upon available biomarker information are one of the most common adaptations in biomarker adaptive designs [149].

CARA procedures are sometimes (incorrectly) referred to as minimisation procedures or dynamic allocation; the methods referred to with these names are very different in their goals and nature. For example, some CARA procedures have been proposed altering the randomisation (similar to RAR) [150] while other methods do not do so in a randomised fashion determining allocation probabilities based solely on covariates [151–153]. Some CARA procedures are designed to minimise imbalances on important covariates only [150, 151] while other methods have an efficiency goal, being designed to minimise the variance of the treatment effect in the presence of covariates [154]. Finally, some CARA rules will aim to assign the largest number of patients to the best treatment while accounting for patients’ differences in biomarkers [155].

**Example** The BATTLE [156] trial is a prospective, biopsy-mandated, biomarker-based, adaptively randomised [157] study conducted in 255 pre-treated lung cancer patients. Initially, 97 patients were randomised equally to four arms: erlotinib, vandetanib, erlotinib plus bexarotene, or sorafenib, based on relevant biomarkers. Then, for the remaining 158 patients, the allocation probabilities were adapted using a CARA procedure. This procedure used a Bayesian adaptive algorithm: the data from the first 97 patients were assessed giving a prior distribution (describing the likely values of the effect of the treatment) for disease control rate (DCR, the primary endpoint) in each biomarker group; this prior distribution was continuously updated using the accumulating data as more patients were observed giving a posterior distribution (describing the likely values of the effect of the treatment having combined information from the prior distribution and the available trial data) for DCR in each biomarker group; upon recruiting, any new patient to the trial their randomisation was governed by the currently available data using posterior distribution. Results include a 46% 8-week disease control rate (primary endpoint) and evidence of an impressive benefit from sorafenib among mutant-KRAS patients.

**Advantages/disadvantages** Because of the similarity to RAR, advantages and disadvantages of CARA designs are very similar. The main advantage being that they allow flexibility, introducing balance, efficiency, or ethical goals according to what might be more relevant. In addition, CARA designs make assumptions about the model for biomarker interactions for patients in the trial and thus are further sensitive to these assumptions.

#### Population enrichment

**Fig. 4:**
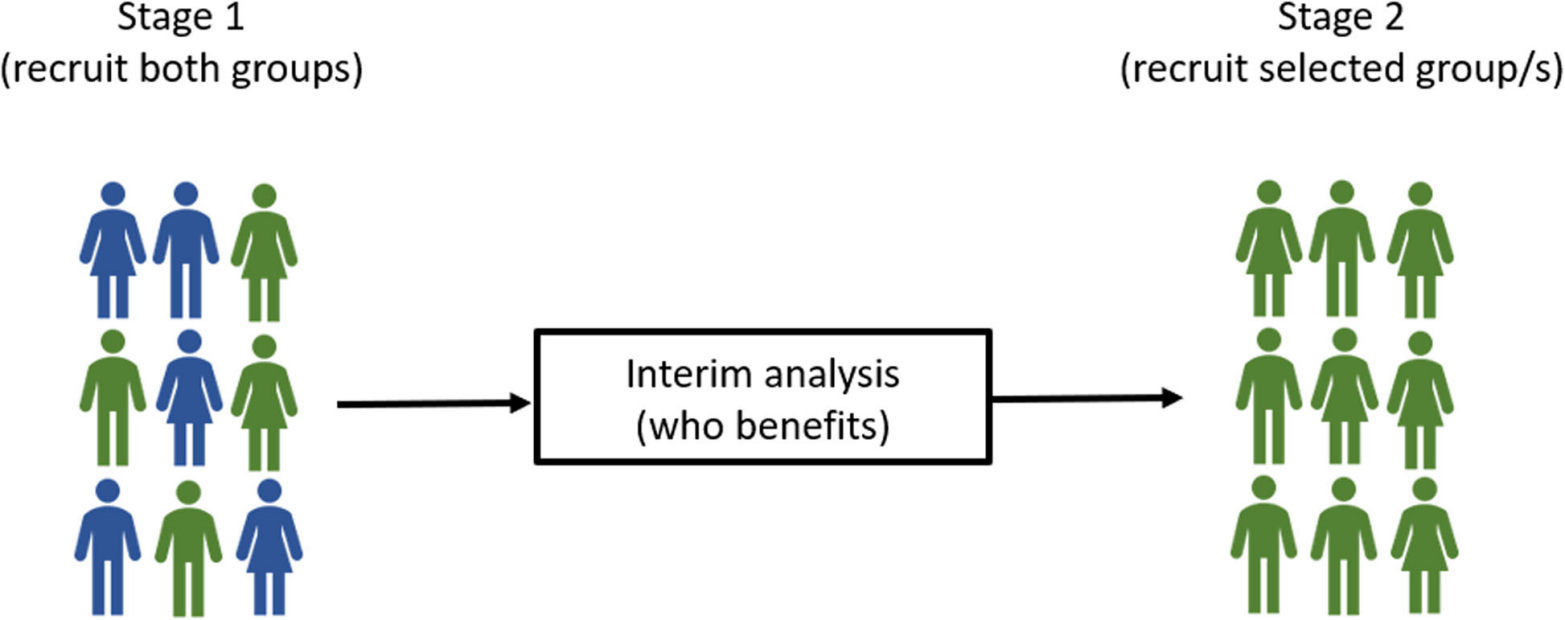
An adaptive enrichment design examining 2 sub-groups

Population enrichment designs are useful when biomarker-defined sub-groups are known before the trial commences. With uncertainty about which populations should be recruited, they select which sub-populations to recruit from for the remainder of the trial based on data available at the interim analysis. Figure 4 illustrates how an adaptive enrichment design can progress. In a non-adaptive trial, the study team must make this sub-population selection before the trial begins. Population enrichment designs are typically planned with one of two goals in mind: target the sub-population where patients receive the greatest benefit, or stop recruiting from sub-populations where the treatment may not provide a benefit.

Flexible hypothesis testing methods [101, 102, 158] preserve the statistical integrity of the trial while allowing freedom in the selection of sub-populations. The decision-making methodology is key in population enrichment with several approaches, both Bayesian [159–161] and classical techniques being applied [162, 163].

**Example** TAPPAS [164, 165] is a trial of TRC105 (an antibody) and pazopanib versus pazopanib alone in patients with advanced angiosarcoma. The study identified two sub-groups, those with cutaneous advanced angiosarcoma and those with non-cutaneous advanced angiosarcoma. There was an indication of greater tumour sensitivity to TRC105 in the cutaneous sub-group. The primary endpoint for this study was progression-free survival, with an initial sample size of 124 patients to be followed until 95 events (progression or death) have been observed.

A population enrichment design was used where the data monitoring committee was able to recommend one of three pre-planned actions at the interim analysis: continue as planned with the full population (recruiting 124 patients followed until 95 events in total), continue with the full population and an increase in sample size and progression-free survival events (recruiting 200 patients followed until 170 events in total), and continue with only the cutaneous sub-group, thereby enriching the study population (recruiting 170 patients followed until 110 events in total). This decision-making procedure followed the promising zone approach [166], where the choice about which option would be followed is based upon the probability of rejecting the null hypotheses given the data available at the interim analysis.

The study recruited from the full population throughout, as dictated by the data observed at the interim analysis. In total, 128 patients were recruited (close to the targeted recruitment of 124), with 64 patients randomised to each the experimental treatment and the control. It was concluded that TRC105 did not demonstrate activity when combined with pazopanib.

**Advantages** Population enrichment designs recruit fewer patients from sub-groups that do not benefit from the treatment focusing on those patients who receive a benefit. When there is uncertainty about which sub-groups benefit from the new treatment, the adaptive method can offer an improvement over non-adaptive alternatives. It is able to offer the benefit of increasing the sample size in one sub-group without sacrificing the opportunity to test the new treatment in all patients before the observation of any patients. In addition, population enrichment offers a compromise between the possible fixed designs (for example recruiting only from one of the two sub-groups, or the full population). This is appealing where there is disagreement between which (sub)group(s) should be recruited.

**Disadvantages** Computing optimal decision rules and evaluating overall trial performance are non-trivial typically requiring some form of simulation [160], increasing the workload in setting up the trial. While possible changes to eligibility criteria during the course of a trial add challenges to the operational planning of patient recruitment.

#### Summary

In this section, we have discussed designs suitable for phase II and phase III of clinical development with a focus on a biomarker-guided approach to treatment. Despite active research in this area, these methods appear to have the lowest uptake at the time of writing, although with the drive towards personalised healthcare they are likely to become increasingly relevant.

The designs discussed target pre-defined sub-populations allowing formal testing for efficacy of an experimental treatment in biomarker-defined sub-populations. Of note, these methods assume these biomarker groups are well defined, with little work on how to properly account for this should this assumption be violated [167]. Beyond being structured to allow formal analysis of sub-groups, there is also a large ethical benefit to these designs, exposing as few patients as possible to treatments from which they may not receive a benefit.

Should no such pre-defined biomarkers be available, one might consider adaptive signature designs [168, 169], also referred to as biomarker adaptive designs [149, 170, 171]. They aim to identify and use predictive biomarkers [170] during the trial. They help to improve the chances of identifying patients who will benefit from the treatment, while still providing accurate treatment effect estimates; this maximises the use of the trial data. However, the identification of predictive biomarkers adds a large amount of complexity.

Umbrella [172] and basket trials [173] make use of more detailed biomarker information. Broadly speaking, a basket trial examines a single experimental treatment in multiple sub-types of a single biomarker, while an umbrella trial may consider mutiple biomarkers each with a specific treatment.

### Does the treatment work?

In this section, we consider treatments late in the development cycle, typically phase III (although viable in phase II also), where we wish to show conclusively that a novel treatment is an improvement over the current standard of care. A conventional aim at this stage of development is to be efficient, both in terms of the time that is required to conduct the trial and the number of patients; the methods that follow attempt to make the trial more efficient while also ensuring that the overall probability of a successful outcome is maintained or increased. Methods aiming to be efficient in recruiting the correct number of patients have both an operational and ethical benefit, ensuring patients are not subject to an experimental treatment for a trial that is underpowered (i.e. has a low chance of detecting a meaningful effect) or when their contribution to the overall result is not required.

#### Group sequential designs

**Fig. 5:**
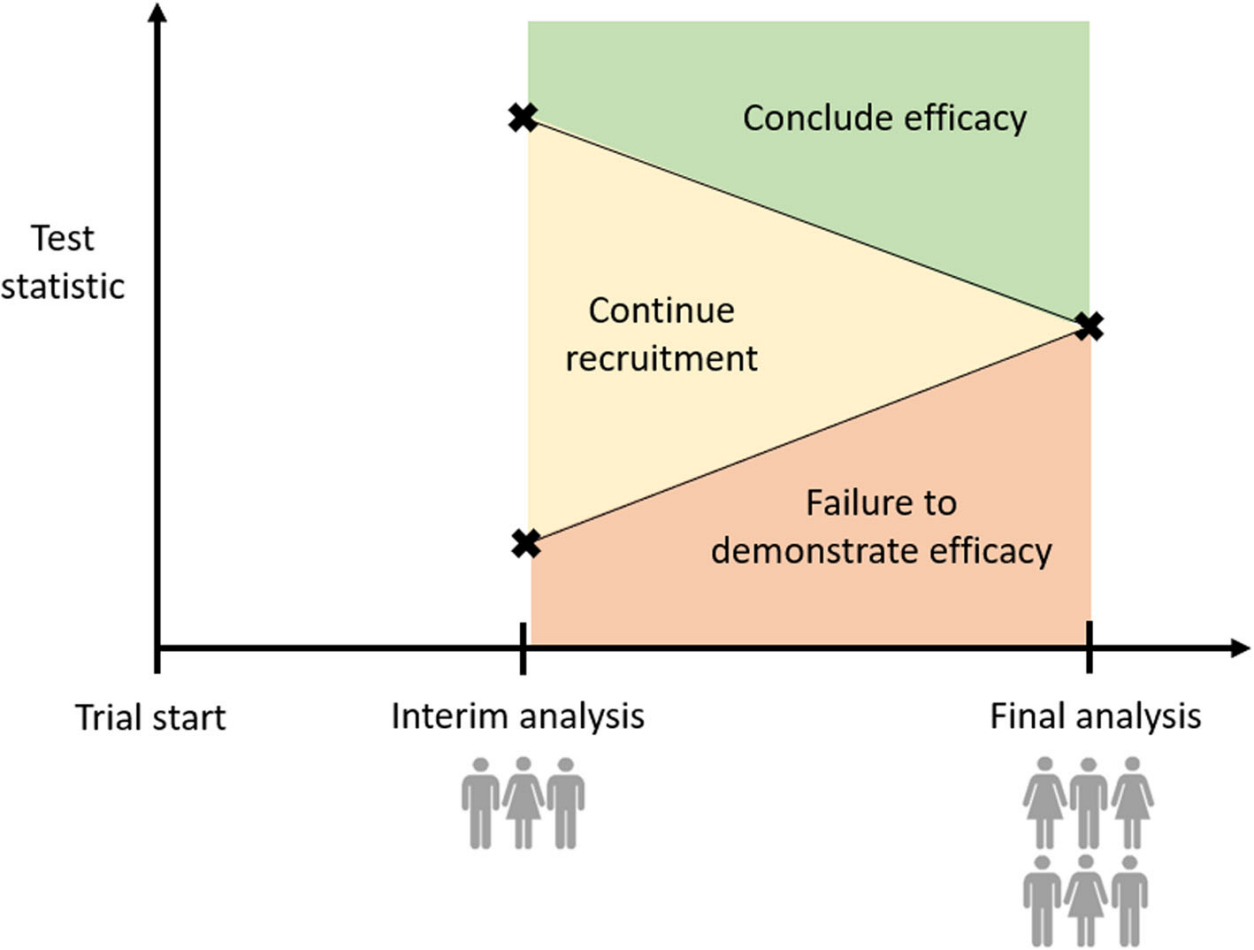
Demonstration of stopping boundaries in a 2-stage group sequential design

Group sequential designs are the most widespread [174] of the adaptive designs we consider. They differ from a more traditional phase III clinical trial in that one or more pre-planned interim analyses may be used to assess efficacy; if there is strong evidence the experimental treatment is superior to control, or indeed that both have the same effect, then the trial may be terminated early as depicted in Fig. 5.

To enable early stopping of the trial while maintaining control of the type I error rate, group sequential trials use pre-defined stopping rules. At each interim analysis, the current data for the experimental and control arms are compared to construct a test statistic. If the test statistic is sufficiently high/low, the trial is stopped early for efficacy/lack of a demonstrated benefit of the treatment; if neither criterion is met, the trial continues to the next interim or final analysis. Several approaches to the definition of stopping rules for group sequential designs have been proposed [5, 175–177]. Jennison and Turnbull [178] provide a comprehensive guide to group sequential designs and their application. Whitehead [179] describes the implementation of group sequential methods using SAS; in addition, the gsDesign [180] and optGS [181] packages in R are open source alternatives allowing the construction and analysis of group sequential designs.

**Example** The INTERCEPT trial [182, 183] was a randomised, double-blind trial in patients with acute myocardial infarction. The trial used a group sequential design to achieve 80% power to detect a 33% between-group difference in cumulative first event rate of cardiac death, non-fatal reinfarction, or refractory ischaemia. Interim analyses were conducted after enrolment of 300 patients, and at intervals of roughly 300 patients thereafter. The stopping boundaries were constructed using a double triangular test [177].

Recruitment was stopped after the third interim analysis as the pre-specified 33% between-group difference for the primary endpoint would likely not be observed. In total, the trial recruited 874 patients. A total of 430 patients were randomised to 300 mg oral diltiazem once daily, and 444 patients were randomised to placebo initiated within 36–96 h of infarct onset, and given for up to 6 months.

**Advantages** Group sequential designs reduce the expected sample size of the trial compared to a traditional trial design with a fixed sample size. This is due to the possibility the trial will stop early due to strong evidence that the treatment either is or is not effective. For O’Brien-Fleming [175] boundaries, this reduction is typically around 15% compared to a fixed design [55]. Group sequential designs are optimal in terms of minimising the expected sample size [178]. Thus, any other design aiming to reduce the expected sample size can only perform as well as the group sequential option. In addition, with group sequential methods being widespread [174], their implementation is likely to be more familiar to regulators and ethics committees.

**Disadvantages** The opportunity for early stopping requires careful definition of stopping boundaries, and there is a cost to this adjustment. While the expectation is for the design to reduce the sample size by stopping early, it is possible that the group sequential design will recruit more patients than a non-adaptive method would have (when no early stopping criterion is met), usually by approximately 10% [55]. Due to uncertainty about the length of the trial before it commences, logistics and planning are more complex than for a traditional fixed sample design.

#### Sample size re-assessment

Sample size re-assessment (also known as sample size re-estimation or sample size re-calculation) seeks to ensure an appropriate sample size for the trial despite uncertainty about key design parameters (such as the variance in the observations) by re-assessing the required sample size during the trial. Typically, the re-assessment focuses on the estimation of design parameters at an interim analysis to re-calculate the sample size in order to achieve, for example, a desired conditional power [184–186] (the probability of rejecting the null hypothesis given the currently available data). Usually, this allows an increase but not a decrease to the sample size, often with a limit on what maximum sample size is possible, as large increases to the sample size in this way can be inefficient [187]; in practice, if the estimated sample size exceeds the pre-set maximum, then recruitment is usually stopped.

Sample size re-assessment may be either blinded or unblinded [188], while maintaining the statistical integrity of the trial. In unblinded sample size re-estimation, interim analyses are conducted based on unblinded trial data; that is, the statistician performing the interim analysis will know which participants are in which trial arm. Updated estimates of parameters related to the sample size are used to re-assess the sample size for the remainder of the trial [189–191]. Conversely, blinded sample size re-estimation only makes use of the combined trial data from all treatment arms [184, 185, 192, 193]. Alterations to the sample size in this way have a minimal impact on the type I error rate, even without formal adjustment [193]. The suitability of blinded or unblinded re-assessment will depend on the parameters that require re-estimation.

The sample size re-assessment must be conducted before the originally planned conclusion of the trial. That is, it must not be used where a trial has completed and failed to reject the null hypothesis. Indeed, it should not be used at all with the intention of recovering a significant result. Equally severely reducing the number of patients to be recruited using sample size re-estimation can be problematic, where it may inflate the probability of falsely rejecting the null hypothesis [194]. Note also that it can be counterproductive to make changes to a trial in progress based on estimates of the treatment effect from the trial itself, as small or no treatment effect results in increased sample sizes so that less useful treatments require more resource. In such a case, a group sequential design where the interim analyses are planned to correspond to different treatment effects of interest (e.g. using an optimistic effect at first interim, moderate effect at second, and minimally relevant effect at final interim) is likely a better choice.

**Example** Hade et al. [195] discuss a sample size re-assessment in a randomised trial in breast cancer where the primary outcome is disease-free survival. Women were randomised either to immediate surgery in the next 1–6 days, which was expected to be in the follicular phase of the menstrual cycle, or to scheduled surgery during the next mid-luteal phase of the menstrual cycle. Based on primary analysis by log-rank test with a target of 80% power, with 5% two-sided type I error rate, to detect a hazard ratio (HR) of 0.58 in favour of scheduled surgery, this required 113 events. With accrual time of 2 years and 4 additional years of follow-up and a 2–3% loss to follow-up, the initial study design planned to randomise 340 women.

The HR of 0.58 was felt to be optimistic based on available information and sources external to the trial. A blinded sample size re-assessment using the available data increased the sample size by 170 patients, to a total of 510 randomised patients (with a required 175 events) in order to target a revised HR of 0.65.

**Advantages** The main aim of sample size re-assessment is to ensure that the trial recruits an appropriate number of patients. Sample size re-assessment designs are not as complex as many other adaptive methods, allowing the trial to be planned more quickly. The fact that both unblinded and blinded methods are available means that sample size re-assessment can be applied in many different settings. There is an upward trend in the use of sample size re-assessment in clinical practice, and as these designs become more widespread, it will become increasingly easy to put them forward.

**Disadvantages** This is a method with few drawbacks. There is a small additional burden at the interim analysis to properly estimate the required sample size for the remainder of the trial, which requires appropriate expertise in the case of both blinded and unblinded interim analyses. Most of the practical issues that a sample size re-assessment design may bring (time constraints or securing sufficing funding in advance) are similar to those faced when using other adaptive designs, while further concerns may be raised from extending the trial beyond the originally planned end. An example is the comparability of patients recruited early and those recruited late to a modified trial [196].

#### Summary

These methods are the most widespread of the adaptive designs we have considered [174], to the extent group sequential trials may even be considered a standard approach. The group sequential framework forms a foundation for many other adaptive methods due to its preservation of the integrity of the trial results.

The fact that such methods are so well established in practice shows promise for the use of the adaptive methods discussed in this paper. Despite the complexity, these methods are sufficiently well-known in the trials community with software support and common practices established allowing their implementation. The other methods discussed throughout this work share many similarities in terms of methodological complexity while each has their own advantages/disadvantages, but these are not so far removed from those that have been overcome for group sequential designs so as to make overcoming these hurdles an impossibility.

## Conclusions

Research into adaptive designs has become more prevalent across all stages of clinical development, although this increase is not necessarily reflected by their uptake in practice. The suitability of adaptive methods depends largely on the clinical question being addressed. We have presented four key clinical questions for which adaptive designs may be of use across a wide range of disease areas, study settings, and endpoints. For each possible design, there are advantages and disadvantages with some key themes: increase in efficiency of the design in terms of the expected number of patients or a clear benefit in understanding the question of scientific interest, clear ethical advantages to ensuring the right patients are given the best available treatment whenever possible, and the key disadvantage is the additional burden, both in planning the trial and the interim analyses. Importantly, while the adaptive methods can be highly effective when used in the correct scenario, an adaptive method is not always the best choice [197], so careful consideration must be taken before their use.

At the design stage of any trial (adaptive or not), some design assumptions must be made. These will influence the performance of the trial and inadequate assumptions can lead to a sub-optimal design. With the additional complexity of many adaptive designs, there are more assumptions to be made and it is critical that these are well understood by the trial team to consider the impact of these choices, although for a corresponding fixed sample design many of these assumptions must be made anyway. As noted in the “Summary” of the “Does the treatment work?” section, many such problems have been well worked out for group sequential designs and are not insurmountable for the other designs discussed. Communication and establishing a common practice between the methodology and trial community will be key in seeing the wider spread application of such methods.

Regulatory bodies are increasingly recognising the desire for the use of adaptive designs and accepting their use although it is recommended regulators be engaged early in the process whenever using any novel methodology [55, 198]. Funding bodies are also increasingly comfortable with the use of adaptive designs; the TAILoR trial discussed in the ‘Multi-arm multi-stage’ section appears as a case study on the National Institute for Health Research website [199]. Additionally, new reporting guidance for adaptive designs [200] has recently been published to facilitate uptake further.

We have not been exhaustive in our discussion of adaptive designs, focusing on the key designs to answer the most common questions of clinical interest. Seamless designs [101, 102, 201], which we have not discussed in detail, use similar adaptive design methodology to combine phases of clinical development: designs may be inferentially seamless, where data from the earlier stage are incorporated into the overall trial results; operationally seamless, avoiding any break in recruitment between the stages of the development process but excluding data from earlier stages from the final analysis of the latter; or both. There are many motivations for conducting seamless designs [202], making it an active area of research [203].

For many years, one major obstacle to the use of adaptive designs in practice has been the lack of suitable software to aid both the design and conduct of trials. This issue is increasingly being tackled by those researching the methods, with many open source packages available for the design and analysis of adaptive methods some of which have been cited in this work. For example rpact [204] is an R package that assists in the design and analysis of confirmatory clinical trials. In addition, there is a steep learning curve to the implementation of such designs; training courses are becoming increasingly available to address this.

From a methodology standpoint, there are some further issues that go beyond the level of detail we have discussed that should be considered when proposing an adaptive design, for example potential information leakage or the introduction of bias [205, 206]. The Practical Adaptive and Novel Designs and Analysis (PANDA) toolkit [207] is under development at the time of writing and will be an online resource that addresses and explains broader issues in the use of adaptive designs.

Despite the challenges in the design and analysis of an adaptive trial, we believe that under the right circumstance, the benefits introduced by the increased flexibility clearly outweigh these issues.

## Data Availability

Not applicable.
